# Monomeric adiponectin increases cell viability in porcine aortic endothelial cells cultured in normal and high glucose conditions: Data on kinases activation

**DOI:** 10.1016/j.dib.2016.08.007

**Published:** 2016-08-10

**Authors:** Elena Grossini, Serena Farruggio, Fatima Qoqaiche, Giulia Raina, Lara Camillo, Lorenzo Sigaudo, David Mary, Nicola Surico, Daniela Surico

**Affiliations:** Department of Translational Medicine, University East Piedmont “A. Avogadro”, Azienda Ospedaliera Universitaria Maggiore della Carità, corso Mazzini 36, Via Solaroli 17, Novara, Italy

## Abstract

We found that monomeric adiponectin was able to increase cell viability in porcine aortic endothelial cells (PAE) cultured both in normal and high glucose condition. Moreover, in normal glucose condition monomeric adiponectin increased p38MAPK, Akt, ERK1/2 and eNOS phosphorylation in a dose- and time-dependent way. Also in high glucose condition monomeric adiponectin increased eNOS and above kinases phosphorylation with similar patterns but at lower extent. For interpretation of the data presented in this article, please see the research article “Monomeric adiponectin modulates nitric oxide release and calcium movements in porcine aortic endothelial cells in normal/high glucose conditions” (Grossini et al., in press) [1].

**Specifications Table**

**Table:** 

Subject area	*Biology*
More specific subject area	*Cardiovascular system, Physiology*
Type of data	*Figures*
How data was acquired	*MTT Assay (Life Technology, Monza, Italia; Italy) and a spectrometer Victor Multilabel plate (PerkinElmer, Waltham, MA, USA) for cell viability; ECL (PerkinElmer) and Versadoc (BioRad, Segrate, Italy) for protein quantification*
Data format	*Analyzed*
Experimental factors	*Porcine Aortic Endothelial cells (PAE) were maintained in high and normal glucose conditions. A 30 mM concentration of culture fluid containing d-glucose was applied to the high glucose group. Furthermore, cells were treated with monomeric adiponectin (0.3 ng, 3 ng, 30 ng, 100 ng)*
Experimental features	*Cells were cultured at 37 °C with 5% CO_2_ in normal and high glucose conditions in order to mimic the stress conditions of hyperglycemia in diabetic patients*
Data source location	*University East Piedmont, Novara, Italy*
Data accessibility	*Data are presented in this article*

**Value of the data**

• The data add information about the protection exerted by monomeric adiponectin in normal/high glucose conditions in PAE.
• The data highlight the different involvements of kinases activation in the effects of monomeric adiponectin in PAE cultured in above conditions.
• The data may represent the starting point of further research about the response of endothelial cells to monomeric adiponectin in terms of cell viability and the related mechanisms of action.

## 1 Data

**Fig. 1:**
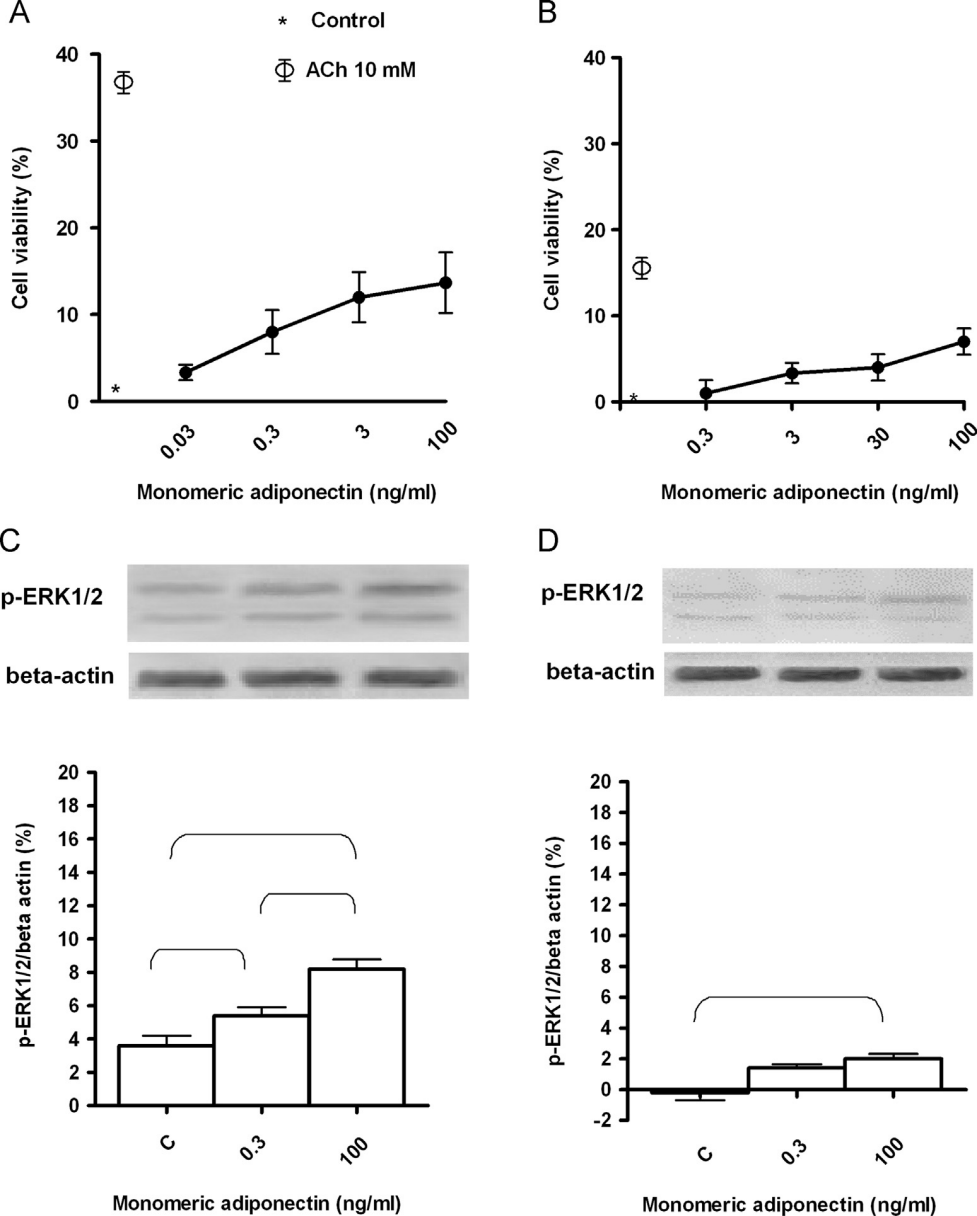
The effects of monomeric adiponectin on cell viability (A, B) and p-ERK1/2 (C, D) in PAE. In A and C, normal glucose condition, in B and D, high glucose condition. In A, ACh: acetylcholine. In C and D, an example of lanes of p-ERK1/2 and densitometric analysis of five different experiments for each protocol are shown. C: control. p-ERK1/2: phosphorylated ERK1/2. The results are means±SD of five independent experiments for each experimental protocol. Parentheses indicate significance between groups (*P*<0.05).

**Fig. 2:**
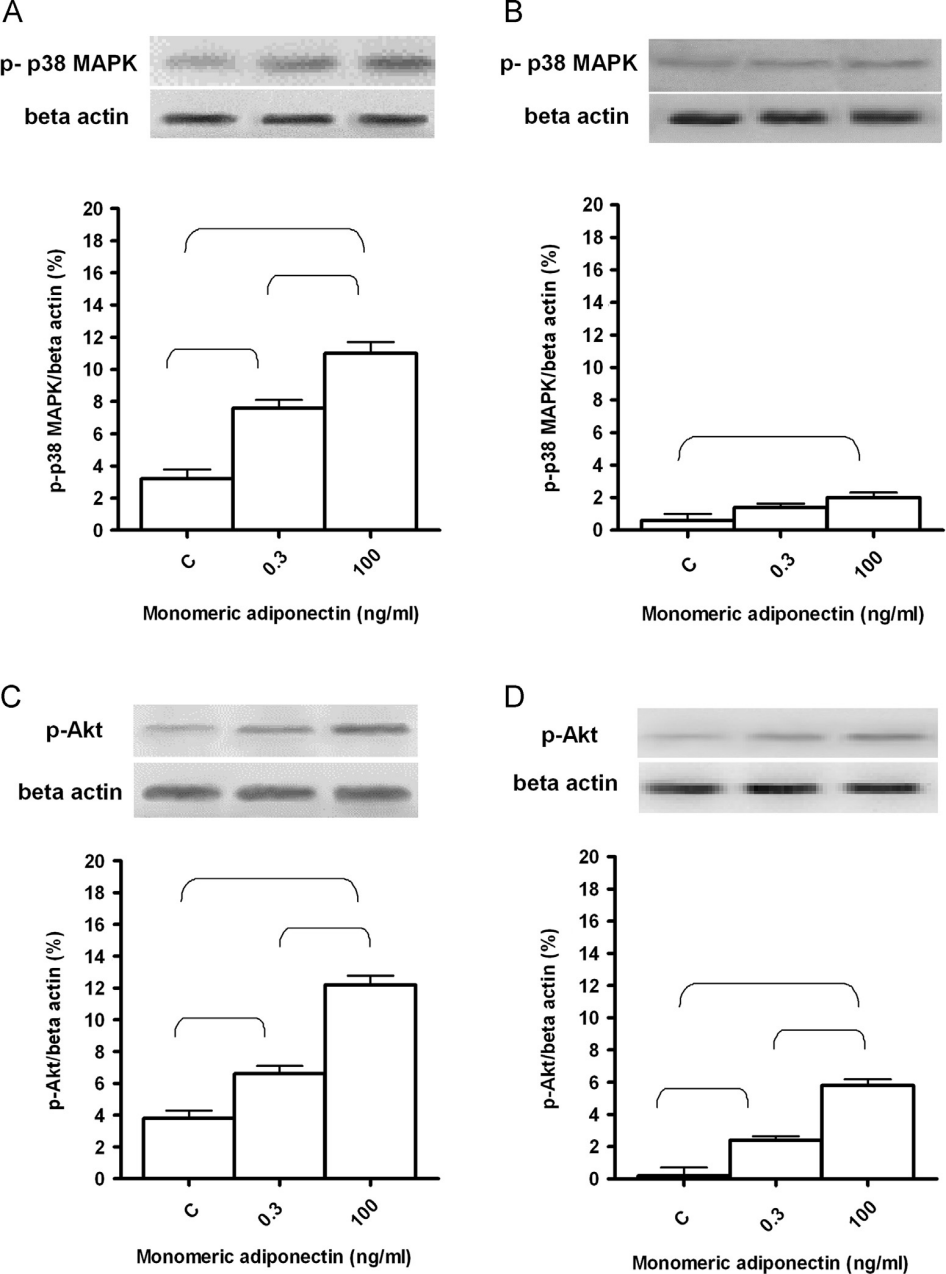
The effects of monomeric adiponectin on p38MAPK (A and B) and Akt (C and D) activation in PAE. In A, normal glucose condition, in B, high glucose condition. An example of lanes and densitometric analysis of five different experiments for each protocol are shown. C: control. p-p38MAPK: phosphorylated p38MAPK. p-Akt: phosphorylated Akt. Parentheses indicate significance between groups (*P*<0.05).

**Fig. 3:**
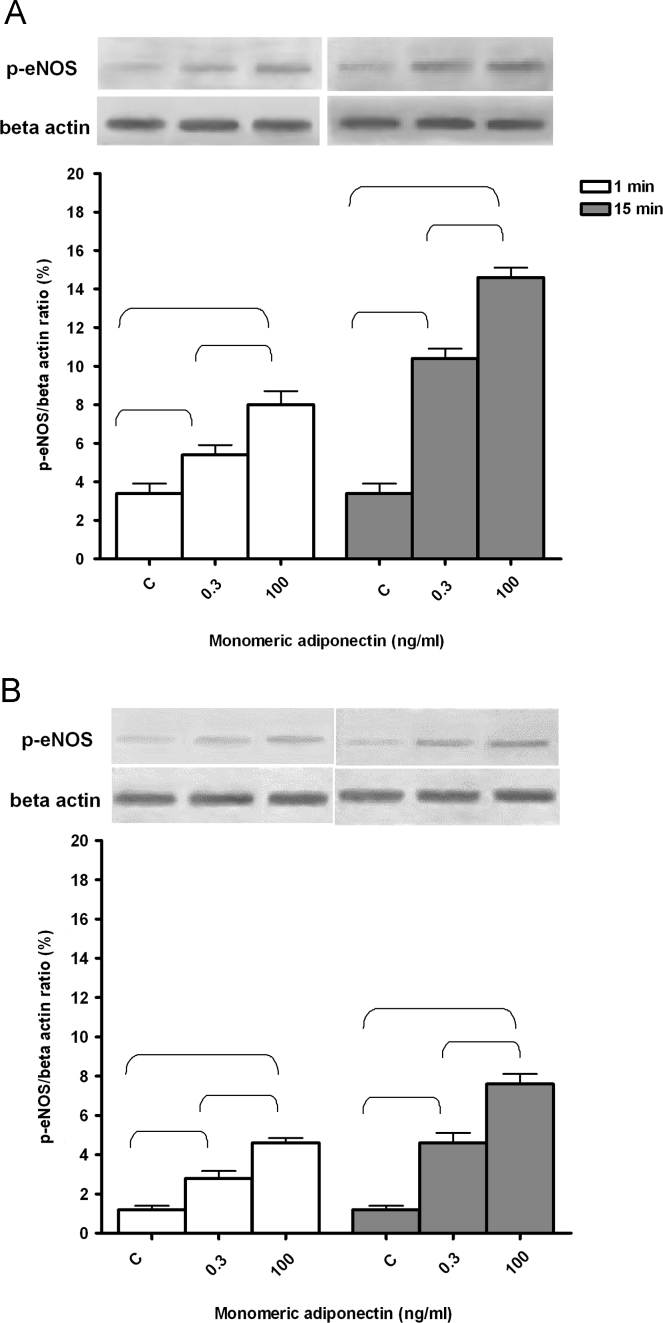
The effects of monomeric adiponectin on eNOS activation in PAE. In A, normal glucose condition, in B, high glucose condition. An example of lanes and densitometric analysis of five different experiments for each protocol are shown. C: control. p-eNOS: phosphorylated eNOS. Parentheses indicate significance between groups (*P*<0.05).

Monomeric adiponectin was able to increase cell viability of PAE cultured both in normal and glucose medium (Fig. 1; *P*<0.05). In addition, a grading was found in ERK1/2, p38MAPK, Akt and eNOS phosphorylation in response to monomeric adiponectin in normal glucose condition (Fig. 1, Fig. 2, Fig. 3). A similar pattern was found in PAE cultured in high glucose medium, although p38MAPK and ERK1/2 phosphorylation, in particular, was markedly lower (Fig. 1, Fig. 2, Fig. 3).

## 2 Experimental design, materials and methods

### 2.1 Culture of PAE

The experiments were performed in high and normal glucose conditions. A 30 mM concentration of culture fluid containing d-glucose was applied to the hyperglycemic group [1].

### 2.2 Cell viability

To determine cell viability, the in vitro Toxicology Assay Kit MTT Based (Life Technologies Italia, Monza; Italy) was used, as previously described [1], [2], [3], [4], [5]. PAE were treated with monomeric adiponectin (0.3 ng/ml, 3 ng/ml, 30 ng/ml, 100 ng/ml; Sigma) for 15 min. Some cell sample were administrated acetylcholine chlorohydrate (10 mM, for 15 min; Sigma). Details are added in Supplemental material.

### 2.3 Kinases activation

Western blot analysis was performed in PAE at ~90% confluence in a 100 mm dishes in DMEM 0% FBS and red phenol (Sigma), as previously described [1], [2], [3], [4], [5]. PAE, cultured in normal and high glucose conditions, were stimulated with monomeric adiponectin (0.3 ng/ml, 100 ng/ml; Sigma) for 15 min. For eNOS activation studies, monomeric adiponectin was administrated for 1 and 15 min. Following antibodies were detected: anti phospho-Akt (p-Akt; Cell Signalling Technologies, Beverly, MA), anti phospho-ERK1/2 (p-ERK1/2; Cell Signalling Technologies), anti phospho-p38MAPK (p-p38MAPK; Cell Signalling Technologies), anti phospo-eNOS (p-eNOS; Santa-Cruz Biotechnology, Inc, CA, USA). Details are added in Supplemental material.

## 3 Statistical analysis

All data were recorded using the Institution׳s database. Statistical analysis was performed by using STATVIEW version 5.0.1 for Microsoft Windows (SAS Institute Inc., Cary NC, USA). Data were checked for normality before statistical analysis. All the results obtained were examined through one-way ANOVA followed by Bonferroni *posthoc* tests. All data are presented as means±SD of five independent experiments for each experimental protocol. A value of *p*<0.05 was considered statistically significant.

## Figures and Tables

**Fig. S1:**
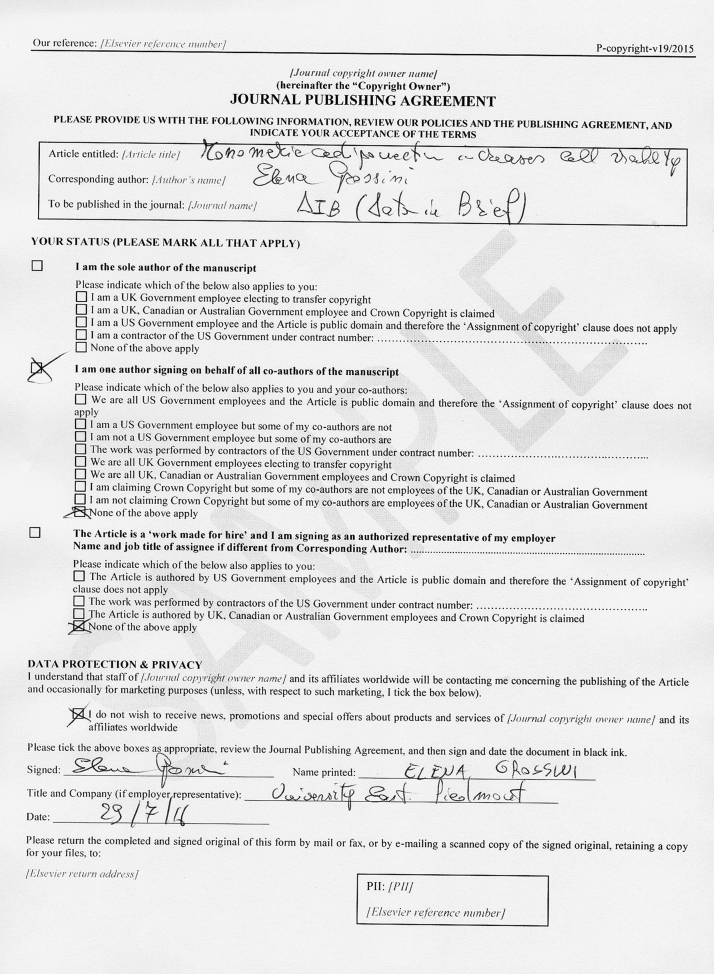
.
