# Supplementary material for: Monomeric adiponectin increases cell viability in porcine aortic endothelial cells cultured in normal and high glucose conditions: Data on kinases activation
Source: Data Brief. 2016 Aug 10;8:1381–6. doi: 10.1016/j.dib.2016.08.007 (PMC4993859; doi:10.1016/j.dib.2016.08.007)
Supplement: Supplementary file 1 — Supplementary material [file mmc1.doc]

Supplemental Material

*Cell viability (MTT Assay)*

To determine cell viability, the In Vitro Toxicology Assay Kit MTT (Life Technologies Italia, Monza; Italy) was used. PAE maintained in high and normal glucose conditions, were cultured 1×10^4^ in 96-well plates in DMEM, 10% FBS and supplemented with L-glutamine, penicillin-streptomycin, HEPES and incubated overnight (100μl/well). Next day the cells were treated with monomeric adiponectin (0.3 ng, 3 ng, 30 ng, 100 ng; Sigma) for 15 min and acetylcholine chlorohydrate (10 mM; Sigma) for 15 min. After treatments, the medium was removed and fresh culture medium without red phenol and FBS containing the 1% 3-[4,5-dimethylthiazol- 2-yl]-2,5-diphenyl tetrazolium bromide (MTT) dye was added in 96-well plates containing the cells and incubated for 2 h at 37 °C in incubator. Thereafter, the medium was removed and MTT Solubilization Solution in equal volume to the original culture medium was added and mixed until the complete dissolution of formazan crystals. Cell viability was determined by measuring the absorbance through a spectrometer (BS1000 Spectra Count, San Jose, CA) and cell viability was calculated by comparing results with control cells (100% viable).

*Kinases activation (WESTERN BLOTTING)*

Cell lysates (30 μg protein each sample) dissolved in Laemmli buffer 5X, boiled for 5 min, were resolved in 10% sodium dodecyl sulfate polyacrylamide gel electrophoresis (SDS-PAGE) gels (Bio-Rad Laboratories, Hercules, CA, USA) and after electrophoresis, were transferred to polyvinylidene fluoride (PVDF) membranes (Bio-Rad Laboratories), which were incubated overnight at 4 °C with specific primary antibodies: anti phospho-Akt (p-Akt; 1:1000; Ser473, Cell Signalling Technologies, Beverly, MA), anti phospho-ERK1/2 (p-ERK1/2; 1:1000; Thr202/Tyr204, Cell Signalling Technologies), anti phospho-p38MAPK (p-p38MAPK; 1:1000; Thr180/Tyr182, Cell Signalling Technologies), anti phospo-eNOS (p-eNOS; 1:1000; Ser1177, Santa-Cruz Biotechnology, Inc, CA, USA). The membranes were washed and then incubated with horseradish peroxidase-coupled goat anti-rabbit IgG (Sigma), peroxidase-coupled rabbit anti-goat IgG and horseradish peroxidase-coupled goat anti-mouse IgG (Sigma) for 45 min and were developed through a non radioactive method using Western Lightning Chemiluminescence (PerkinElmer Life and Analytical Sciences, Waltham, MA, USA). Phosphorylated protein expression was calculated as a ratio towards β-actin (1:5000; Sigma) detection.
